# In vivo biochemical assessment of cartilage with gagCEST MRI: Correlation with cartilage properties

**DOI:** 10.1002/nbm.4463

**Published:** 2020-12-22

**Authors:** Sander Brinkhof, Razmara Nizak, Sotcheadt Sim, Vitaliy Khlebnikov, Eric Quenneville, Martin Garon, Dennis W.J. Klomp, Daniel Saris

**Affiliations:** ^1^ Department of Radiology University Medical Center Utrecht Utrecht the Netherlands; ^2^ Department of Orthopaedics University Medical Center Utrecht Utrecht the Netherlands; ^3^ Biomomentum Inc. Laval Quebec Canada; ^4^ MIRA Institute for Biomedical Technology and Technical Medicine University of Twente Enschede the Netherlands; ^5^ Department of Orthopaedics, Mayo Clinic Rochester Massachusetts United States

**Keywords:** cartilage, gagCEST, osteoarthritis, 7 T

## Abstract

To assess articular cartilage in vivo, a noninvasive measurement is proposed to evaluate damage of the cartilage. It is hypothesized that glycosaminoglycan chemical exchange saturation transfer (gagCEST) can be applied as a noninvasive imaging technique as it would relate to electromechanical indentation and GAG content as measured with biochemical assays. This pilot study applies gagCEST MRI in total knee arthroplasty (TKA) patients to assess substantially damaged articular cartilage. The outcome was verified against electromechanical indentation and biochemical assays to assess the potential of gagCEST MRI. Five TKA patients were scanned on a 7.0 T MRI with a gagCEST sequence. Articular resurfacing cuts after TKA were obtained for electromechanical and biochemical analyses. The gagCEST MRI measurements on the medial condyle showed a moderate correlation with the GAG content, although sensitivity on the lateral condyle was lacking. Additionally, a strong negative correlation of gagCEST MRI with the electromechanical measurements was observed in the regression analysis. Correlation of gagCEST MRI with electromechanical measurements was shown, but the correlation of gagCEST MRI with GAG content was not convincing. In conclusion, gagCEST could be a useful tool to assess the GAG content in articular cartilage noninvasively, although the mismatch in heterogeneity requires further investigation.

## Graphical abstract

This pilot study applies gagCEST MRI in vivo in five total knee arthroplasty patients to assess substantially damaged articular cartilage. GagCEST MRI measurements show a moderate correlation with GAG content on the medial condyle and a strong correlation with electromechanical measurements. In conclusion, gagCEST could be a useful tool to assess the GAG content in articular cartilage noninvasively, although the mismatch in heterogeneity requires further investigation.

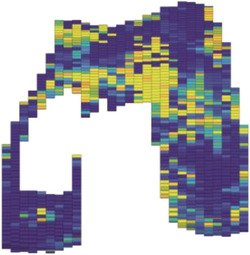

**ABBREVIATIONS USED**
DMMB: dimethylmethylene blue
GAG: glycosaminoglycan
gagCEST: glycosaminoglycan chemical exchange saturation transfer
ICC, intraclass correlation coefficient; ICRS: International Cartilage Regeneration and Joint Preservation Society
MT: magnetization transfer
NOE: nuclear Overhauser effect
OA: osteoarthritis
QP: quantitative parameter
TKA: total knee arthroplasty

## 1 INTRODUCTION

Early‐stage cartilage damage is characterized by changes in the mechanical integrity and biochemical composition of articular cartilage, mainly disorientation of the collagen fibers in the superficial zone of cartilage and loss of glycosaminoglycans (GAGs) throughout the cartilage. The GAG content is of specific interest because its hydrophilic capacity helps the joint to withstand mechanical load. Loss of GAG content leads to a decrease in stiffness of the articular cartilage and therefore a lower ability to withstand normal loads.^1^, ^2^ These changes can occur after trauma, during aging and/or during the course of degenerative joint diseases such as osteoarthritis (OA). The regenerative abilities of cartilage are virtually nonexistent, which makes early identification of cartilage damage essential for successful treatment.^3^, ^4^ To understand the development of cartilage damage and degeneration and identify possible treatment markers, measurements of these changes in biochemical composition are vital. These measurements can be carried out ex vivo, such as histological assessments, biochemical assays or biomechanical testing measuring the biochemical composition in cartilage. However, they are invasive and destructive in nature and cannot be used in vivo.

Subtle changes in cartilage composition, in either healthy or damaged cartilage, can be visualized with quantitative MRI. The quantitative assessment of GAG content is a biomarker for early cartilage damage. Chemical exchange saturation transfer (CEST) MRI is a promising technique for noninvasive evaluation of this GAG content in articular cartilage.^5^, ^6^, ^7^, ^8^ CEST can be used to monitor the GAG content in articular cartilage noninvasively, based on the chemical exchange of the labile hydroxyl protons of GAG with the bulk water. Recently, we have been able to show within a feasibility study in vivo at 7 T that gagCEST MRI can clearly discriminate between healthy cartilage and damaged cartilage, with stable and reproducible measurements.^8^

Assessment of articular cartilage with quantitative MRI is not only necessary in diagnosis but is also of importance for treatment planning and monitoring. GagCEST could aid orthopedic surgeons in the noninvasive preoperative and postoperative assessment of cartilage. During surgery, orthopedic surgeons rely on their arthroscopic blunt probe to evaluate the cartilage, which is subject to interpretation.

Electromechanical indentation is a technique that can be used to assess the cartilage nondestructively in vivo. The probe is similar to an arthroscopic blunt probe but instrumented with an array of 37 microelectrodes on the surface of its hemispherical indenter. These microelectrodes measure streaming potentials induced through indentation, which, due to their molecular origins, are particularly sensitive to functional integrity of the collagen network and GAG content.^9^, ^10^ This technique has been shown as correlated with destructive cartilage (quality) assessments such as histology, biochemistry and mechanical indentation.^11^, ^12^

Implementation of a noninvasive measurement for quantitative assessment of cartilage in vivo has an added clinical value to evaluate the damage, plan treatment or follow up after treatment. GagCEST could be a valuable method to assess the cartilage for all these purposes, whereas electromechanical indentation can be used during surgery to precisely localize the lesion to be treated.^10^, ^12^ This pilot study aims to apply gagCEST MRI in vivo in total knee arthroplasty (TKA) patients to assess the articular cartilage. The outcome is consequently related with electromechanical indentation and biochemical assays to assess the potential of gagCEST MRI in clinical practice.

## 2 MATERIALS AND METHODS

### 2.1 Study workflow

This pilot study adheres to the Declaration of Helsinki, was approved by the institutional ethics review board (Medisch Ethische Toetsingscommissie Utrecht, protocol number 15/672D) and written informed consent was obtained from the participants. Five patients (aged 56‐68 years, two males and three females) scheduled to undergo TKA were included in this study. Patients were selected within the specialized knee clinic of the University Medical Center Utrecht. The experiments were carried out on a 7.0 T whole body scanner (Achieva; Philips Healthcare, Best, the Netherlands) using a volume transmit coil and a wrap‐around 32‐channel receiver knee coil (MR Coils BV, Zaltbommel, the Netherlands). Participants were scanned up to 1 day before their surgery. A 3D gagCEST sequence was used with 19 offsets (range between −900 Hz/−3 ppm and 900 Hz/3 ppm, with normalization offsets of ±100 KHz), with an acquisition time of 6 minutes 59 seconds and a resolution of 1 x 1 x 3 mm^3^. The 3D gagCEST sequence entailed a presaturation train of 20 sinc‐shaped pulses (B1 = 2 μT, pulse length = 20 ms, duty cycle = 70%). The readout parameters were as follows: five‐shot turbo field echo (TFE), TFE factor of 370, SENSE factor of 2, TR/TE/FA = 2.75 ms/1.4 ms/5 degrees, field of view 140 x 150 x 135 mm^3^ (covering the whole knee) and inter‐shot T1 recovery time of 2 seconds. The sequence used is presented in an earlier work.^8^

Femoral resurfacing cuts were collected for each TKA patient during surgery. The entire surface of these femoral weight‐bearing resurfacing cuts was mapped manually ex vivo using a benchtop version of the electromechanical probe (Biomomentum Inc., Laval, QC, Canada), measuring streaming potentials induced from the compression of articular cartilage.^11^ The output is the quantitative parameter (QP, A.U.) corresponding to the number of microelectrodes in contact with the articular cartilage when the sum of the streaming potentials is 100 mV. The QP is thus inversely proportional to the electromechanical response of the articular cartilage. This indicates that a low QP corresponds to strong electromechanical properties and high load‐bearing characteristics, while a high QP is defined by signs of cartilage degeneration.^11^ Recently, a new output of the electromechanical probe has been developed. This electromechanical grading system allows assessment of cartilage quality quantitatively,^13^ where the electromechanical QP (defined on a 0‐37 scale) was translated into an electromechanical grade (defined on a 0‐4 scale), a grade analogous to the International Cartilage Regeneration and Joint Preservation Society (ICRS) grade. A position grid was overlaid through camera‐registration software (Mapping Toolbox; Biomomentum Inc., Laval, QC, Canada) to guide through the manual electromechanical assessment of femoral cuts. The hemispherical indenter of the electromechanical probe was then rapidly compressed onto the cartilage surface (<1 second) and the electromechanical QP and electromechanical grade were recorded at each position of the grid.

**FIGURE 1:**
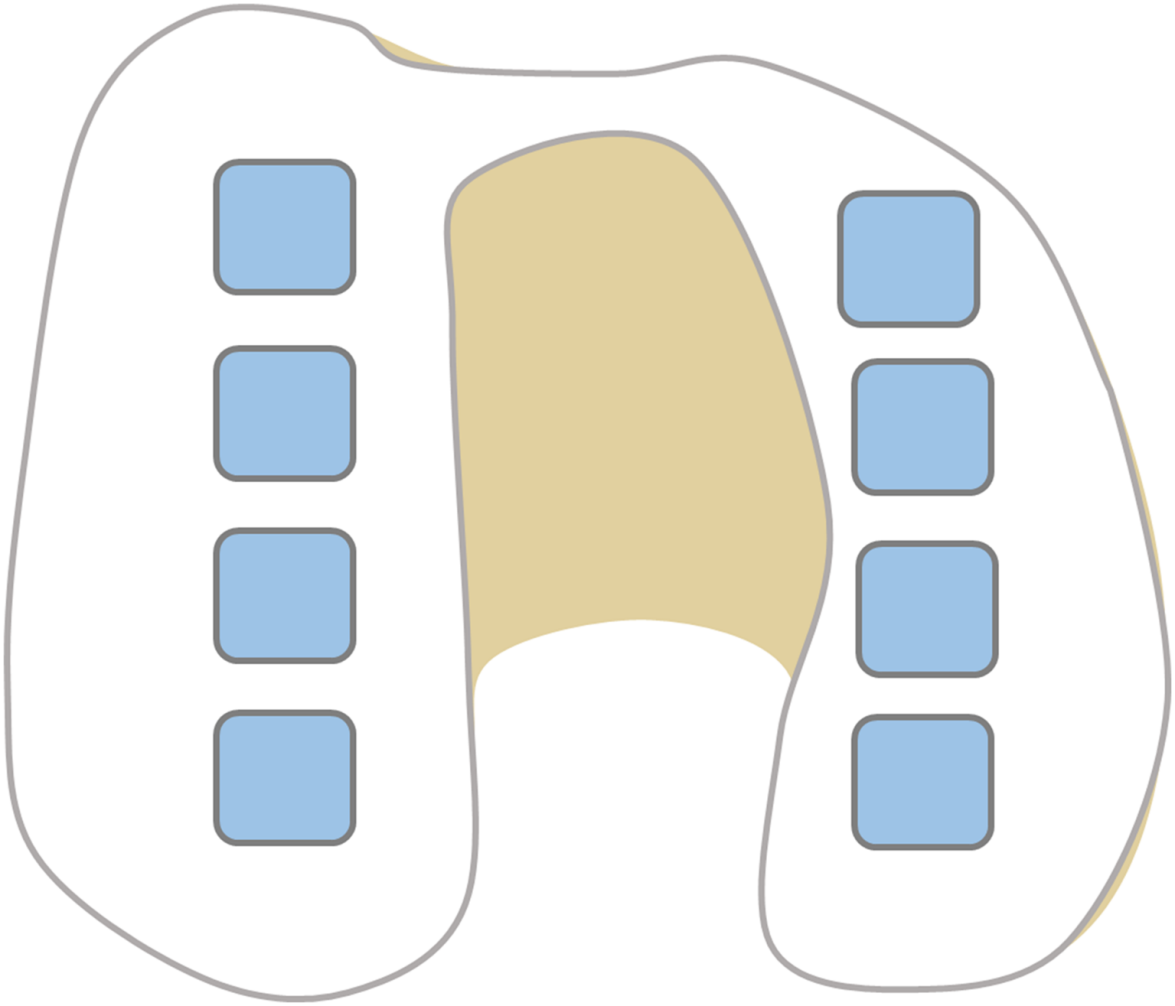
Axial view of the knee. Overview of the cartilage samples (blue squares). Eight cartilage samples (squares of 1 cm^2^) were taken for DMMB assay, four in each femoral condyle

**FIGURE 2:**
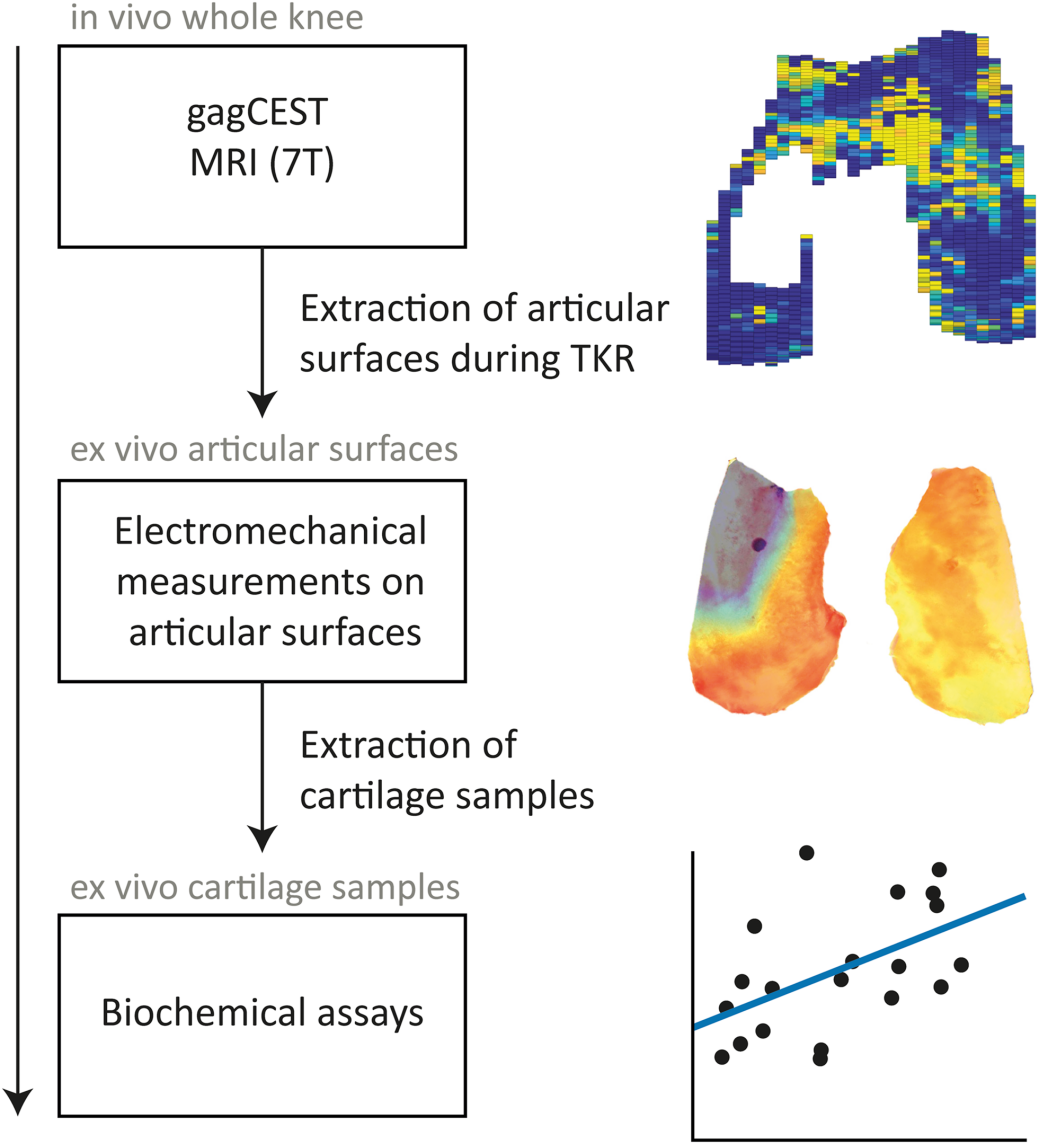
Overview of study procedures

After electromechanical indentation mapping, the cartilage samples (1 x 1 cm^2^) were taken from the resurfacing cuts and processed for biochemical analyses, by means of a dimethylmethylene blue (DMMB) assay to quantify the GAG weight in the samples.^14^ The locations for biochemical analyses (blue squares) are shown in the graphical overview of femoral articular cartilage in Figure 1. Each distal condyle had four standardized sample locations for DMMB analyses, adding up to eight per knee, totaling up to 40 samples (although not every sample location had enough cartilage to be included for further analyses). Additionally, DMMB analyses were also carried out on two standardized sample locations for each posterior condyle, adding up to 20 extra samples in total. These samples were specifically used within the analyses of DMMB values versus gagCEST values. All samples were weighted before papain digestion solution (250 μg/mL papain, Sigma‐Aldrich) was added for sample digestion by incubation overnight at 60°C. The digested samples were diluted in PBS and stained with DMMB staining solution. The total amount of GAG was measured photospectrometrically in duplicate by dividing the extinction at 525 nm by the extinction at 595 nm with shark chondroitin‐6‐sulfate (Sigma‐Aldrich) as a standard. The GAG concentration per wet weight was used in the data analysis. A graphical overview of the study procedures is presented in Figure 2.

### 2.2 Data analysis

The gagCEST MRI data were manually coregistered to the biochemistry locations using the presurgical MRI planning for patient‐specific TKA planning (Visionaire; Smith & Nephew, Inc., Memphis, TN, USA). Using this information, the locations of the biochemical measurements and electromechanical measurements were registered and correlated with the gagCEST MRI values. The MRI data were corrected for B0 inhomogeneities (making use of B0 correction with WASSR,^15^ using the five offsets around 0 Hz to center the peak) and consequently normalized, whereafter a three‐pool Lorentzian fit was carried out. The normalized amplitude of the GAG pool was hereafter used as the outcome measure. Within this three‐pool Lorentzian fit a separate fit was carried out for GAG, water and magnetization transfer (MT) effects. The GAG pool was fitted in the range of 0.6 to 1.2 ppm. Possible effects of collagen in the articular cartilage reside in the MT effect and by fitting this separately there was no interference with the GAG pool. Because the choice of frequency offsets was dedicated to the GAG pool, there were not enough frequency offsets to assess other pools such as nuclear Overhauser effect (NOE) or NH. The registration and subsequent data analysis were performed in MATLAB (R2016b, MathWorks, Natick, MA, USA) with in‐house–developed processing scripts.

### 2.3 Statistical analysis

The cartilage samples were assumed to be independent, since we analyzed a measurement for cartilage quality assuming it to be independent for patient‐specific characteristics. The cartilage samples were therefore used independently from one another in the analysis. The analysis was carried out in a three‐step approach, where the first step was a general performance assessment, as to whether gagCEST can observe differences in GAG content between medial and lateral condyles. To achieve this, average gagCEST values were calculated per condyle with their respective ranges. The second step was an assessment on a sample‐by‐sample basis, where the data were divided between medial and lateral samples. The correlation between gagCEST values and GAG content as measured by DMMB was assessed by a linear mixed model, with patient as a random effect. The third and final step was a regression analysis between the electromechanical gradings (which are sampled on a finer grid, with about 110 data points in total) and the respective gagCEST values on those corresponding locations.

## 3 RESULTS

**FIGURE 3:**
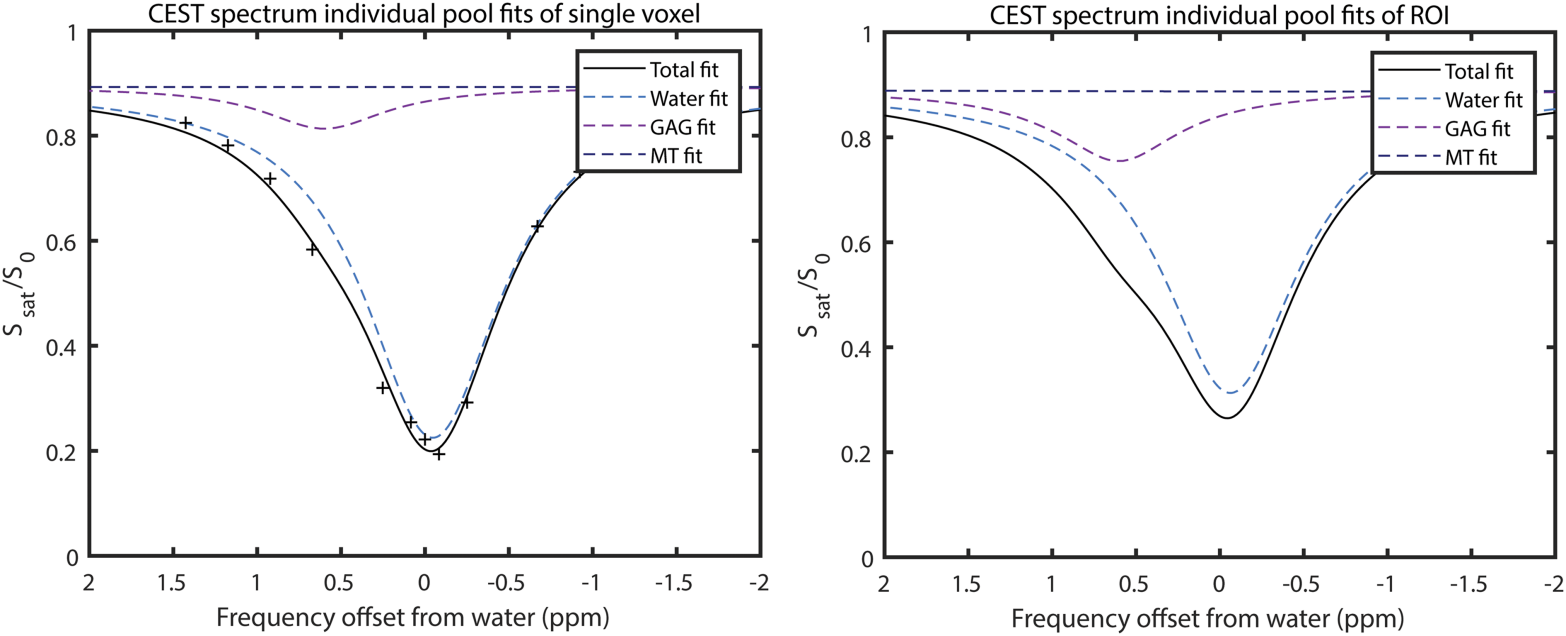
Example fits of a single voxel (left pane, including single data points) and ROI (right pane) of T01

**FIGURE 4:**
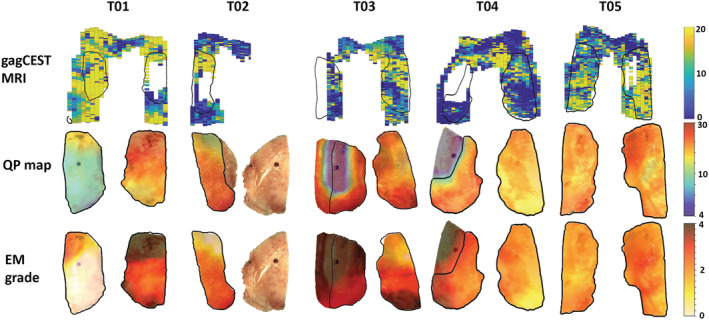
gagCEST MRI maps (upper row), articular resurfacing cuts with QP maps superimposed (middle row) and articular resurfacing cuts with electromechanical grade (EM) maps superimposed (bottom row). The outlines of the articular resurfacing cuts are shown on the gagCEST MRI map with the black lines. Medial condyles: T01 – right side, T02 – right side, T03 – left side, T04 – left side and T05 – right side

Ten cartilage sample locations were excluded due to complete denudation of the bone, while one sample location had just one voxel in cartilage depth on MRI and thus was also excluded to avoid partial volume effects. Figure 3 shows examples of fits of a single voxel (left) and ROI fit (right). Figure 4 shows gagCEST MRI maps (top row) and their corresponding QP (middle row) and electromechanical maps (bottom row) superimposed on the femoral resurfacing cuts resulting from the electromechanical analyses. As an example, the data of T02 show full‐thickness cartilage loss on the medial condyle (exposed bone) but also very degraded cartilage, which is visible in the gagCEST MRI as dark blue or empty (since there is no cartilage), orange/red in the QP map and red/black in the electromechanical grade map. Some parts of the gagCEST map are empty, which correspond with parts where a complete denudation of bone was present (ie, no cartilage).

**FIGURE 5:**
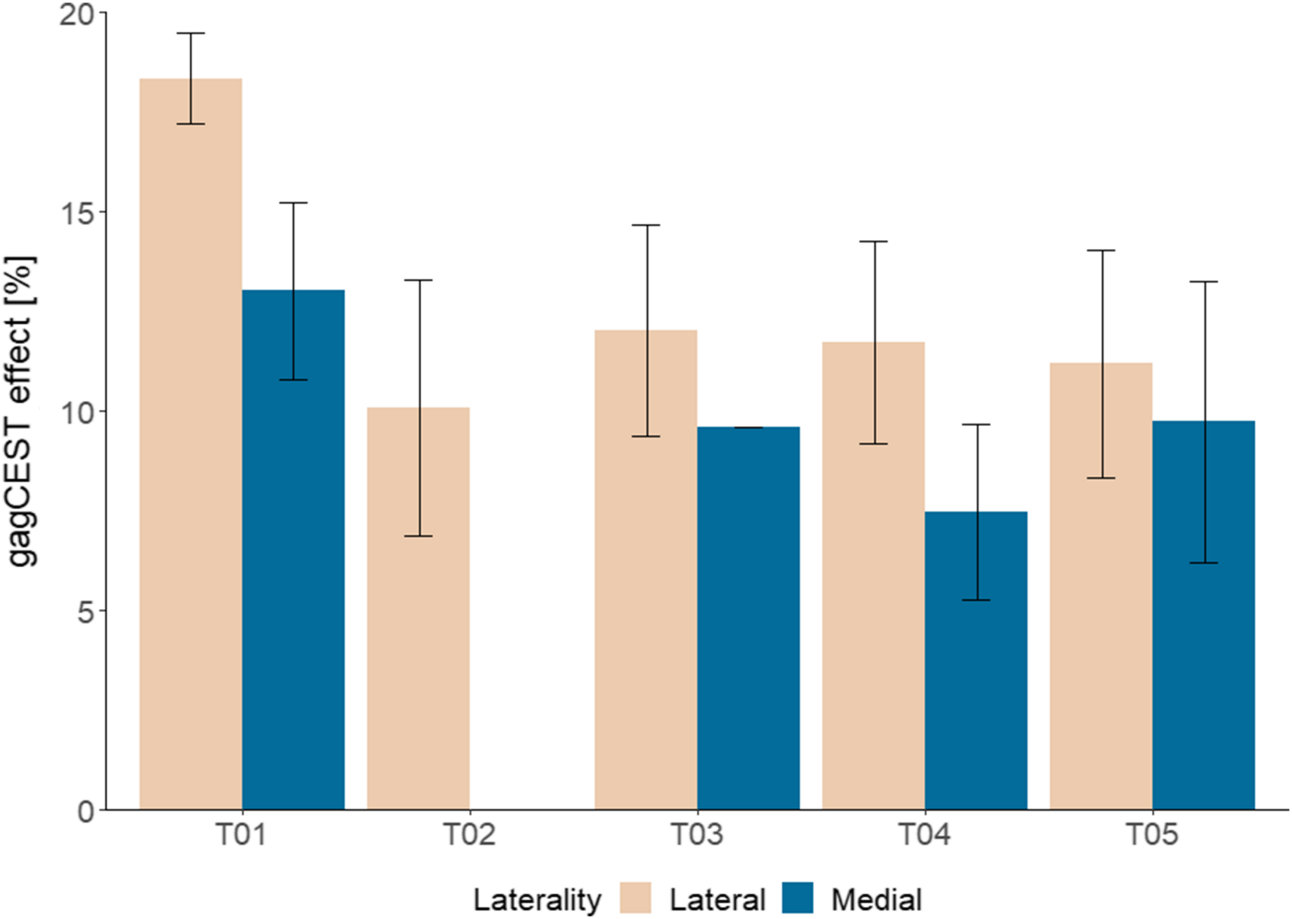
Average gagCEST MRI values per condyle (medial versus lateral) in every patient. Average medial gagCEST effect of 9.95% and average lateral gagCEST effect of 12.41%

**FIGURE 6:**
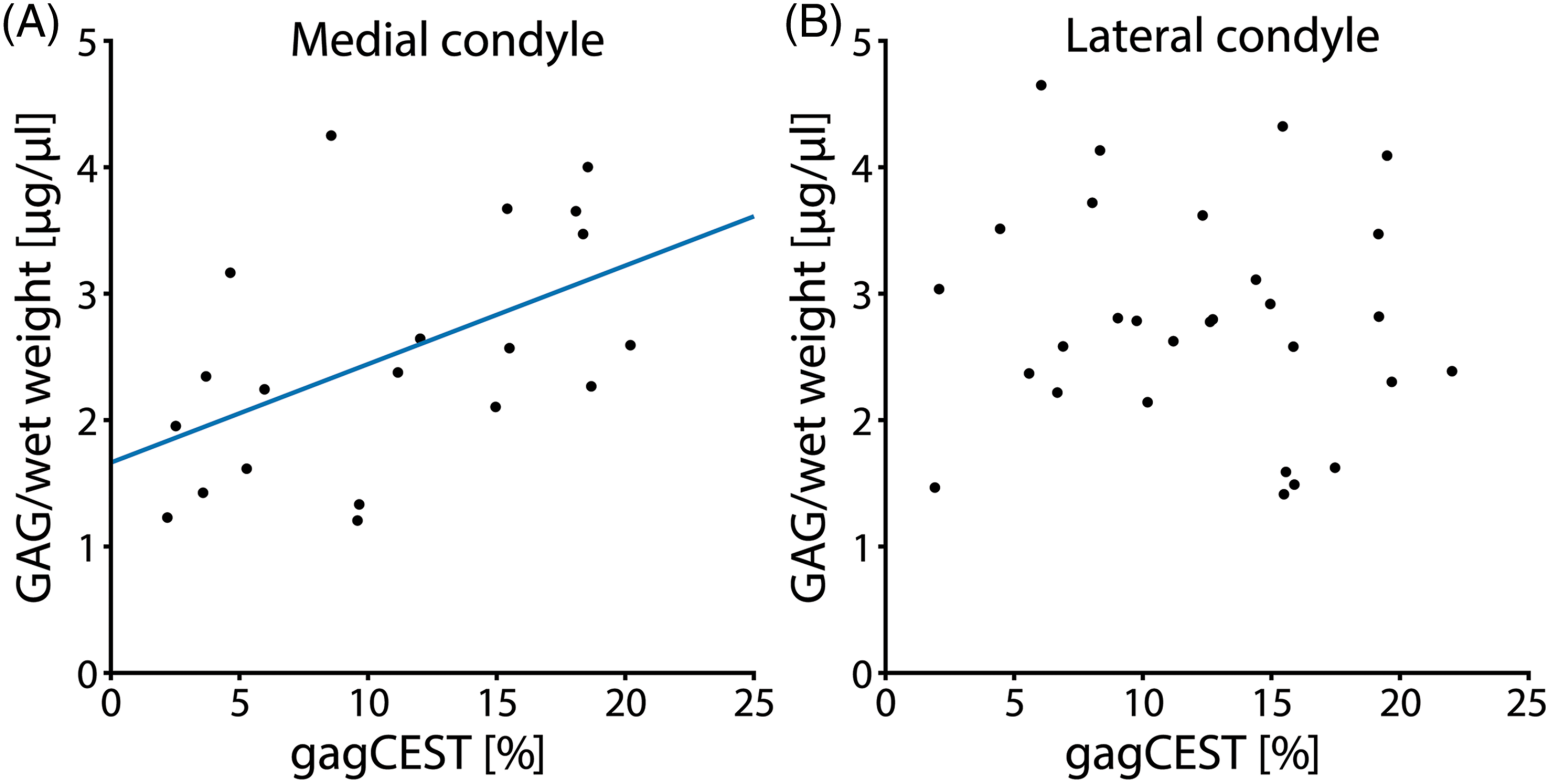
Correlation graphs of GAG concentration as measured by DMMB and gagCEST MRI. A, all the data points of the medial condyle are plotted, including the trendline (r = 0.51, *P* = .05). B, all the data points of the lateral condyle are plotted, which show no clear trend (*P* = .47)

Figure 5 shows the average gagCEST values per condyle, showing a higher gagCEST value in every lateral condyle compared with the corresponding medial condyle (gagCEST effect of 12.41% in the lateral condyle versus 9.95% in the medial condyle; *P* < .05). The complete denudation of the medial condyle is the reason why data are lacking for T02 in Figure 4. This figure shows that the expected trend of a higher gagCEST effect in the lateral condyle compared with the medial condyle holds. Figure 6 shows the correlation of gagCEST with corresponding DMMB values of the medial (A) and lateral (B) condyles. Whereas the medial values show a clear trend towards correlation of gagCEST with GAG per wet weight (*P* = .05), the lateral values do not show this trend (*P* = .47).

**FIGURE 7:**
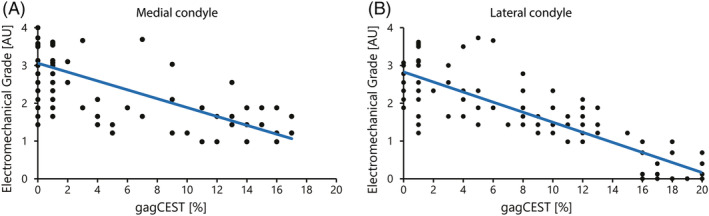
Plot of the electromechanical grade versus gagCEST MRI of each measured data point. A, all the data points of the medial condyle are plotted, including the regression line (r = −0.69, R^2^ = 0.47). B, similar for all the data points of the lateral condyle (r = −0.88, R^2^ = 0.77)

GagCEST MRI is shown to be related with the electromechanical grade in Figure 7. Figure 7 is divided into the medial (A) and lateral (B) data points, showing regression lines for all the data points in the medial and lateral condyle, respectively. Both result in a moderate to strong negative linear relationship (medial: r = −0.69, R^2^ = 0.47; lateral: r = −0.88, R^2^ = 0.77).

## 4 DISCUSSION

This pilot study shows the possibilities of preoperative cartilage quality assessment with gagCEST MRI and intra‐operative cartilage quality assessment with an electromechanical probe. Preoperative assessment with gagCEST on 7 T MRI shows a good correlation with the ex vivo assessment using the electromechanical probe. The regression analysis results in a moderate to strong negative relationship (r = −0.69 for medial samples and r = −0.88 for lateral samples) between gagCEST MRI and electromechanical grade, indicating that gagCEST MRI, just like electromechanical grading, may be useful for accurate biochemical assessment of cartilage.

However, we also observe a lack of sensitivity, which is especially visible in the lateral condyle. There is no correlation between gagCEST MRI and cartilage GAG content per cartilage wet weight (as measured with DMMB) in the lateral condyle. If we compare the results with our earlier feasibility study, excellent test–retest values (an ICC of 0.97) were observed in the lateral condyle, meaning that the signal stability should not lead to errors.^8^ In the same feasibility study we showed that the signal in the medial condyle was also stable and reproducible (an ICC of 0.87). Although we did not perform test–retest measurements in this work, we expect that the spread in data is not due to reproducibility issues, something we assessed in our earlier study. We did not take bias into account, such as T2 relaxation time effects. These effects could result in a signal loss of 5% (based upon a T2 relaxation time in osteoarthritic tibial plateaus of 29.1 ms^16^ and the chosen TE of 1.4 ms). Other confounding effects could be the GAG type in human cartilage, which has been shown in recent work by Einarsson et al^17^ to be of influence in gagCEST measurements.

We assume that the GAG content might be lower in the lateral condyle in this patient group compared with a general elderly population, since these patients suffer from medial OA. These patients often have varus knees (as was the case in four of the five patients included in the current study), which has an influence on the weight‐bearing distribution. Nonweight‐bearing cartilage has lower GAG content,^18^ which therefore might be a spoiling effect in this analysis.

In the process of aging, GAGs are lost in the superficial and middle layers of the cartilage,^19^ and the GAG synthesis in general decreases.^20^ When GAGs are lost in the cartilage and general cartilage volume is retained, a very low DMMB measurement could be the result. GAG quantification by means of DMMB is an absolute measurement of GAG content, although normalized by the wet weight of the sample. This implies that DMMB might not be able to capture the subtle variations in GAG content throughout the depth of the cartilage.^21^ In general, DMMB analyses in OA patients can be difficult to carry out properly. Cartilage is very thin and often some bone will come loose as well, increasing the weight greatly but not increasing the GAG content. The very thin cartilage of OA patients can lead to misinterpretations of the DMMB analyses and/or low sensitivity in gagCEST MRI.

In the medial condyle, a moderate correlation of gagCEST MRI and DMMB‐measured GAG content was observed. Other parameters/factors in the cartilage could interfere with the measured GAG effect by gagCEST MRI. Articular cartilage consists of ~10%‐15% of GAG (wet weight) and 60% of collagen (dry weight), of which the latter could in principle interfere with our gagCEST results.^3^ Within our data analysis, we fit for MT effects, where we expect the effects of the collagen to be visible. The effects of collagen are fitted separately, and therefore minimize its effect on the gagCEST measurements.

Our data do show a lower GAG content measured with gagCEST compared with healthy controls from our previous study.^8^ This is in line with the results of Krishnamoorthy et al, showing that OA patients have a lower GAG content in general on gagCEST MRI.^22^ In addition, gagCEST MRI was also shown to be age‐dependent; the elderly patients included in this study were expected to have a lower gagCEST measurement.^23^ We did observe differences in gagCEST effect size per patient, indicating that the gagCEST effect size might not only be age‐dependent, but could also be dependent on gender and body mass index.^23^, ^24^ In a pilot study such as this one, those effects cannot be considered due to the small number of patients. In a study with a larger population size, these effects should be considered and corrected for wherever possible.

One limitation of this study is the small sample size of severe OA patients, which only enabled assessment of cartilage damage of ICRS grade III and IV. To develop a specific tool that can be used as a biomarker, early cartilage damage (ICRS grade I and II) should also be included.^25^ Another limitation of this study is the manual coregistration of biochemistry locations with the gagCEST MRI and electromechanical measurements. Since every knee has its own shape and curvature, a standard map could not be used. Using the patient‐specific saw guides planning leads to the most optimal results for manual coregistration. Regarding the shape and curvature of the knee, one also has to consider the possible partial volume effects that are present. These effects were even more pronounced in our patient group with OA because of the already thinner cartilage. To minimize the influence of partial volume effects, we chose to exclude data consisting of one voxel in cartilage thickness on gagCEST MRI. Another technical limitation is the choice for the number and location of offsets within the CEST protocol. We chose to opt for 17 offsets (plus two normalization offsets) to be within a clinically applicable scan time. This resulted in the fact that the GAG pool was properly sampled, but other potentially interesting pools such as the NOE or NH pool could not be assessed. Additionally, the inclusion of these pools could theoretically lead to better fitting accuracy.

Higher resolution acquisition could lead to better mapping of spatial variation of the curvatures in cartilage. By using a semi‐continuous wave gagCEST protocol with alternating subsets of amplifiers it is possible to overcome duty cycle limitations and RF signal droop effects to ultimately achieve 1‐mm isotropic resolution.^26^ By using this higher resolution for further work, the sensitivity of gagCEST, especially in regions such as the trochlea, is expected to improve significantly. The maximum duty cycle can also be traded in for a higher in‐plane spatial resolution, which may give insight into laminar differences of GAG content in cartilage depth.

Other MRI methods to target GAGs in articular cartilage include dGEMRIC, T1rho or sodium imaging. Early studies targeting GAG quantification implemented delayed gadolinium‐enhanced MRI of cartilage (dGEMRIC)^27^, ^28^ to measure GAG content indirectly. This imaging protocol relies on the inversely proportional relationship of the negatively charged GAGs with a gadolinium‐based contrast agent in cartilage. An alternative without the need for contrast agents is T1rho, which makes use of a spin‐lock pulse to quantify interactions between motion‐restricted water molecules with their local macromolecular environment. Although this technique has been applied frequently in assessment of articular cartilage, the results seem to vary.^7^, ^29^ Alternatively, sodium (^23^Na) MRI can measure the GAG content by means of measuring the sodium ions in the interstitial fluid in the cartilage.^30^, ^31^, ^32^ Sodium counterbalances the negative charge of the sulfate and carboxyl groups of GAGs, which means that the measurement of sodium and GAG are proportional to one another. We chose to implement gagCEST imaging to have a direct noncontrast alternative to the aforementioned methods.

The electromechanical measurements correlate well with the gagCEST measurements. In patients with OA, GAG content decreases, which leads to a loss of negatively charged molecules residing in the articular cartilage. Less pressure is therefore generated through the compression of articular cartilage, leading to a lower compression‐induced streaming potential resulting in a higher QP value in degraded cartilage. The strong correlation of electromechanical measurements with GAG content measured with gagCEST MRI in this study confirms this hypothesis.

In general, the results of this pilot study demonstrate that noninvasive gagCEST MRI correlates well with the electromechanical properties of articular cartilage. The correlation with GAG content is not convincing, since there is still a lack of sensitivity, mainly in the lateral condyle. This lack of sensitivity warrants further investigation within a larger study setting, given that other parameters do indicate that gagCEST may be promising for application in clinical practice.

## Data Availability

The data that support the findings of this study are available on request from the corresponding author. The data are not publicly available due to privacy or ethical restrictions.
